# Test-retest reliability of selected items of Health Behaviour in School-aged Children (HBSC) survey questionnaire in Beijing, China

**DOI:** 10.1186/1471-2288-10-73

**Published:** 2010-08-10

**Authors:** Yang Liu, Mei Wang, Jorma Tynjälä, Yan Lv, Jari Villberg, Zhouyang Zhang, Lasse Kannas

**Affiliations:** 1 Research Centre for Health Promotion, Department of Health Sciences, University of Jyväskylä, Jyväskylä, Finland; 2 Mass Sports Research Centre, China Institute of Sport Science, Beijing, China; 3 Department of Sport and Health Science, Nanjing Institute of Physical Education, Nanjing, China

## Abstract

**Background:**

Children's health and health behaviour are essential for their development and it is important to obtain abundant and accurate information to understand young people's health and health behaviour. The Health Behaviour in School-aged Children (HBSC) study is among the first large-scale international surveys on adolescent health through self-report questionnaires. So far, more than 40 countries in Europe and North America have been involved in the HBSC study. The purpose of this study is to assess the test-retest reliability of selected items in the Chinese version of the HBSC survey questionnaire in a sample of adolescents in Beijing, China.

**Methods:**

A sample of 95 male and female students aged 11 or 15 years old participated in a test and retest with a three weeks interval. Student Identity numbers of respondents were utilized to permit matching of test-retest questionnaires. 23 items concerning physical activity, sedentary behaviour, sleep and substance use were evaluated by using the percentage of response shifts and the single measure Intraclass Correlation Coefficients (ICC) with 95% confidence interval (CI) for all respondents and stratified by gender and age. Items on substance use were only evaluated for school children aged 15 years old.

**Results:**

The percentage of no response shift between test and retest varied from 32% for the item on computer use at weekends to 92% for the three items on smoking. Of all the 23 items evaluated, 6 items (26%) showed a moderate reliability, 12 items (52%) displayed a substantial reliability and 4 items (17%) indicated almost perfect reliability. No gender and age group difference of the test-retest reliability was found except for a few items on sedentary behaviour.

**Conclusions:**

The overall findings of this study suggest that most selected indicators in the HBSC survey questionnaire have satisfactory test-retest reliability for the students in Beijing. Further test-retest studies in a large and diverse sample, as well as validity studies, should be considered for the future Chinese HBSC study.

## Background

Health behaviour of young people is a global concern. Currently, in China, a large range of problems concerning the health behaviour of the youth is emerging along with changes in lifestyle brought about by rapid economic development and globalization [1,2]. So far, only few national surveys concerning the health behaviour of the Chinese youth have been conducted. In addition to national level research, many studies which investigate a particular health behaviour, or a number of health behaviours and lifestyle traits of young people, have been done by Chinese researchers independently or through a collaborative project with foreign researchers [3-9]. Nevertheless, very few of them can give a comprehensive and comparable portfolio of health behaviour of young Chinese people.

Research exploring children's health behaviours and the factors that influence them are important for the development of effective health education and health promotion programs and policies for young people [10]. Many national and international level studies concerning young people's health behaviour have been conducted in recent decades. The Health Behaviour in School-aged Children (HBSC) study is among the first large-scale international surveys on adolescent health [11]. The participating countries, however, are only within Europe and North America. Since the HBSC study is a tool to examine health behaviour of young people, it is important to seek more international support to examine whether the survey instrument is useful in different continents and cultures. Therefore, for the development of the application of the HBSC study, it is significant and meaningful to expand its boarders in the future to include China, which has the largest population of school-aged children in the world.

Health behaviour is of crucial importance for the adolescents' health and their development [12-18]. It is important that the first step toward understanding young people's health is to obtain abundant and accurate data which represent the prevalence of health behaviour of the young people. Surveys are the most common methodological technique to understand and assess young people's health behaviour, especially in epidemiological studies where the use of a self-report questionnaire is often the only feasible method for the measurement of health behaviour such as physical activity [19]. Therefore, the reliability of the self-report questionnaire measuring health behaviour of adolescents is crucial since the low reliability may tend to mask the real prevalence and important relationships, which adds difficulties or leads to the wrong development of relevant policies, programmes and practices for the young people.

Meanwhile, the test-retest reliability can be influenced by many factors. From the viewpoint of information process of answering questions, two main components of those factors can be distinguished; that the first component is the interpretation or understanding of a question, such as the familiarity of content, complexity and ambiguity of an item, and the second one is the role of memory [20]. Random answers may be found for those items which involve unfamiliar knowledge, or are too complex to understand and therefore yield an uncertain answer, or are ambiguous, leading to variable responses [21]. In addition, it is also understandable that the memory may affect the retest response if the time interval between the test and the retest is short; normally the time interval of test-retest reliability studies is chosen from one week to five weeks. Besides the information processing factors mentioned above, the nature of the item being measured can also affect the test-retest reliability [22]. For instance, the rather stable behaviour, liking smoking, may show higher test-retest reliability than the fluctuated behaviour, such as bullying or injuries.

The reliability of some existing HBSC items have been assessed by a number of countries in recent years, for example, Torsheim and his colleagues investigated the test-retest reliability of 31 selected items in Norway which were used as the indicators in the HBSC study [23]. Later, more studies concerning a specific topic have been done, such as family affluence [24], diet [25], overweight and obesity [26], physical activity [27-29], symptoms [30], reasons for exercise [31], sleep [32] and school environment [33]. In general, the data from those above mentioned studies indicate that most items of the HBSC survey questionnaire had acceptable reliability.

However, more research should be conducted on the survey indicators in different countries and cultures to ensure the continuous improvement of the survey instrument. In order to provide recommendations and conduct revisions for the future Chinese HBSC study, the pilot study using the HBSC 2005/06 survey questionnaire was completed in the Beijing area in 2008. The purpose of this study, therefore, was to examine the test-retest reliability of selected indicators from the HBSC questionnaire measuring physical activity, sedentary behaviour, sleep, and substance use in a Chinese population.

## Methods

### Sampling

This test-retest study is one part of the pilot study for the Health Behaviour and Lifestyle Survey for School-aged Children in Beijing 2008 in which the HBSC 2005/06 survey questionnaire was used. One primary school and one secondary school were randomly chosen in Beijing to conduct the pilot study. Two classes in grade 6 (students aged around 11 years old) and two classes in grade 10 (students aged around 15 years old) were randomly drawn from the two sample schools. All the students (n = 139) in these four classes participated in Test 1. Of those respondents, all the students from one class in grade 6 and two classes in grade 10 completed the questionnaire Test 2. Students from one class in grade 6 did not participate in Test 2 due to the overlap of the school schedule and the survey. No significant difference of the characteristics was found in Test 1 between the class in grade 6 of which students participated in both Test 1 and 2 (n = 44) and the drop out class (n = 44) (Table 1). The final sample for the test-retest study, therefore, consisted of 95 students. The demographic characteristics of respondents are shown in Table 2. The proportion of boys and girls was almost equal in the younger age group, but among the older age group, there were more boys than girls. The mean age of respondents did not differ between boys and girls in either age group.

**Table 1:** Pearson Chi-Square Tests for response of the participants in Test 1 between the participants in both Test 1 and Test 2 (aged 11 years old, n = 44) and the non-participants in Test 2 (aged 11 years old, n = 44)

Items	*P *value
MVPA in the last 7 days	0.35
MVPA in the usual week	0.99
VPA frequency	0.25
VPA time per week	0.67
Watching TV on school days	0.85
Watching TV at weekends	0.94
Playing PC or console games on school days	0.54
Playing PC or console games at weekends	0.36
Using PC on school days	0.34
Using PC at weekends	0.48
Doing homework on school days	0.51
Doing homework at weekends	0.67
When go to bed on school days	0.24
When go to bed at weekends	0.81
When wake up on school mornings	0.17
When wake up at weekends	0.34

**Table 2:** Demographic characteristics of respondents

	Test 1				Test 2				Age (years and decimals) of respondents answered in both tests			
			
	11		15		11		15		11		15	
	n	%	n	%	n	%	N	%	Mean	SD	Mean	SD
Boys	41	46.6	31	60.8	20	45.5	31	60.8	11.79	0.30	15.81	0.34
Girls	47	53.4	20	39.2	24	54.5	20	39.2	11.63	0.37	15.78	0.30
Total	88	100	51	100	44	100	51	100	11.70	0.35	15.80	0.32

### Questionnaire items

The questionnaire used in this study was based on the mandatory and optional questions of the HBSC Protocol for 2001/02 Survey [10] as well as the questionnaire used in the Finnish HBSC Survey in 2006. The questionnaire was firstly translated from English into Chinese by two researchers independently and re-translated from Chinese into English to check the discrepancies by other professional translators. Finally, the questionnaire contained 102 questions and the same questionnaire was used in both the test and the retest. Of those items, 23 items concerning physical activity (4 items), sedentary behaviour (8 items), sleep (4 items) and substance use (7 items) were evaluated in this test-retest study. The detailed information of items and their response alternatives can be found in Table 3.

**Table 3:** The selected items and response alternatives of HBSC survey questionnaire used in test-retest study

Items	Response Alternatives
***Physical Activity***	
1. Over the past 7 days, on how many days were you physically active for a total of at least 60 minutes per day?	*0 day; 1; 2; 3; 4; 5; 6; 7 days*.
2. Over a typical or usual week, on how many days are you physically active for a total of at least 60 minutes per day?	*0 day; 1; 2; 3; 4; 5; 6; 7 days*.
3. OUTSIDE SCHOOL CLASS: How OFTEN do you usually take physical exercise in your free time so that you lose your breath or sweat?	*Daily; 4-6 times a week; 2-3 times a week; Once a week; Once a month; Less than once a month; Never*.
4. OUTSIDE SCHOOL CLASS: How many HOURS a week do you usually take physical exercise in your free time so that you lose your breath or sweat?	*None; Approx. half an hour; Approx. an hour; Approx. 2-3 hours; Approx 4-6 hours; Seven hours or more*.
***Sedentary Behaviour***	
*How many hours a day do you usually do the following things in your free time?*	
5. Watch TV or videos or DVDs on school days	*None; Approx. half an hour; *
6. Watch TV or videos or DVDs at weekends	*Approx. an hour; Approx. 2 hours;*
7. Use a computer for playing games or use console games on school days	*Approx. 3 hours; Approx. 4 hours; *
8. Use a computer for playing games or use console games at weekends	*Approx. 5 hours; Approx. 6 hours; *
9. Use a computer for: chatting online, internet, emailing, homework etc on school days	*Approx. 7 hours or more*.
10. Use a computer for: chatting online, internet, emailing, homework etc at weekends	
11. Spend doing your school homework out of school hours on school days	
12. Spend doing your school homework out of school hours at weekends	
***Sleep***	
13. When do you usually go to bed if you have to go to school in the next morning?	*No later than 21:00; 21:30; 22:00; 22:30; 23:00; 23:30; 24:00; 00:30; 01:00; 01:30; 02:00 or later*.
14. When do you usually go to bed at weekends or during holidays?	*No later than 21:00; 21:30; 22:00; 22:30; 23:00; 23:30; 24:00; 00:30; 01:00; 01:30; 02:00; 02:30; 03:00; 03:30; 04:00 or later*.
15. When do you usually wake up on school mornings?	*No later than 05:00; 05:30; 06:00; 06:30; 07:00; 07:30; 08:00 or later*.
16. When do you usually wake up at weekends?	*No later than 07:00; 07:30; 08:00; 08:30; 09:00; 09:30; 10:00; 10:30; 11:00; 11:30; 12:00; 12:30; 13:00; 13:30; 14:00 or later*
***Risk Behaviour: substance use***	
17. Have you ever smoked?	*Yes; No*.
18. How often do you smoke at present?	*Every day; Every week, but not daily; Less than once a week; I do not smoke*.
19. How many cigarettes, pipefuls or cigars have you smoked until now?	*None; One; Approx. 2-50; More than 50*.
*At present, how often do you drink...?*	
20. Beer	*Daily; At least once a week; At least once a month;*
21. Wine	*Rarely; Never*.
22. Strong liquors	
23. Have you ever had so much alcohol that you have been really drunk?	*Never; Yes, once; Yes, 2-3 times; Yes, 4-10 times;* *Yes, more than 10 times*.

HBSC = Health Behaviour in School-aged Children

### Data collection procedure

The test was administered by one researcher from the China Institute of Sport Science (CISS) and one class teacher from the school during an ordinary class hour. The students were instructed how to fill in the questionnaire by the researcher and they were not informed about the forthcoming retest. Three weeks later the retest was conducted through an identical procedure. All students participating in the test and retest were asked to write their student Identity number on the questionnaire to permit matching the test and retest questionnaires. Student's participation in the test and retest was totally voluntary and the questionnaire, as well as the student Identity number, can only be accessed by the researcher. Students were also informed that only the researcher will read their answers. Verbal consent was sought from all the participants, the head teachers of the classes, and the principle of the school. The test and retest were done at the end of October and at the middle of November in 2008. The study was approved by the ethics committee of CISS and the Research Centre for Health Promotion at the University of Jyväskylä.

### Data analyses

All data from test and retest studies were entered by Epidata 3.1 with double entry and validation and analyzed by Statistical Package for the Social Sciences, version 15.0 (SPSS, Inc., Chicago, Illinois, US). Overall stability rate of items were given by the proportion of subjects showing no response shift on the item between test and retest. The frequency of response shifts of 1, 2 and 3 or more categories were also computed. The test-retest reliability of all selected items were estimated using the single measure of Intraclass Correlation Coefficients (ICC) which were computed as devised by Shrout and Fleiss [34], through case 2 (using a two-way random model with an absolute agreement type), with 95% confidence interval (CI), for all respondents and stratified by gender and age. These values were considered significantly different if their 95% confidence intervals (CIs) did not overlap. According to Landis and Koch [35], the strength of test-retest agreement for ICC is classified as follows: below 0.20 is poor; 0.21 to 0.40 shows a fair agreement; 0.41 to 0.60 indicates a moderate degree of agreement; 0.61 to 0.80 means substantial agreement; and 0.81 to 1 indicates almost perfect agreement. These classifications were used to interpret the results. The items about substance use were evaluated only for the adolescents aged 15 years old due to the absence in this behaviour among 11 years-old respondents.

## Results

**Figure 1:**
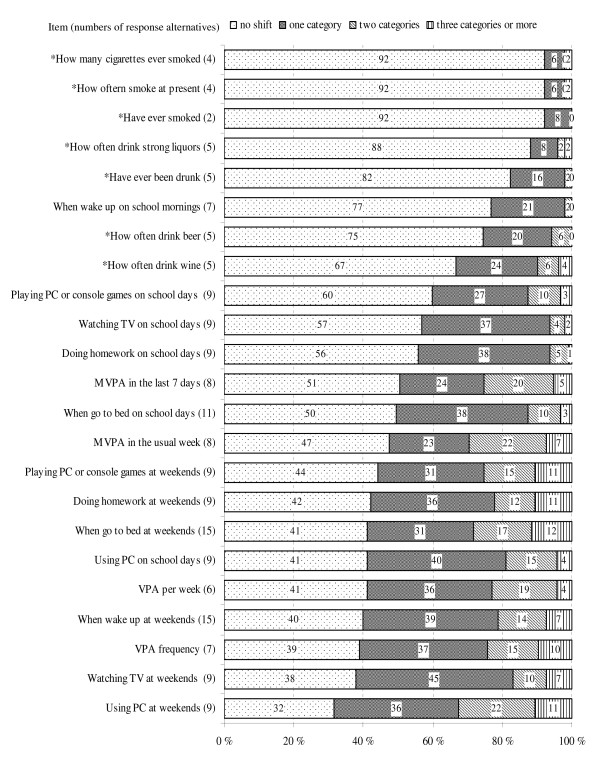
**Frequencies of test-retest shifts on all selected HBSC survey questionnaire items, sorted according to the frequencies of no response shift, descending order (n = 95)**. *Items were only computed for respondents aged 15 years old (n = 51).

The proportions of no response shift between test and retest varied from 32% for the item measuring computer use at weekends, to 92% for the three items on smoking behaviour. At least 68% of the respondents gave an answer in the same or an adjacent category for all selected indicators (Figure 1).

The values of ICC for all respondents were stratified by gender and age. These are shown in Tables 4 and 5. Overall, the values of ICC of the selected items ranged from 0.33 to 0.85, with the lowest value for the item regarding using a computer on school days, and the highest value for items on how many cigarettes ever smoked and pertaining to the question "have you ever been drunk?" Of all the 23 items evaluated in this study, according to Landis and Koch divisions of agreement [35], 6 items (26%) showed a moderate reliability, 12 items (52%) displayed a substantial reliability and 4 items (17%) indicated almost perfect reliability. By gender, the values of ICC varied from 0.19 to 0.96 for girls and 0.42 to 0.85 for boys. The items of the highest and lowest ICC for girls are not consistent with the items for boys. By age groups, ICC ranged from 0.38 to 0.86 for 11 year-old respondents and 0.16 to 0.85 for 15 year-old respondents.

**Table 4:** ICC for HBSC survey questionnaire items about physical activity, sedentary behaviour and sleep by gender and age (n = 95)

	All (n = 95)		Girls (n = 44)		Boys (n = 51)		11 years (n = 44)		15 years (n = 51)	
					
Items	ICC	95% CI	ICC	95% CI	ICC	95% CI	ICC	95% CI	ICC	95% CI
***Physical Activity***										
MVPA in the last 7 days	0.82	0.74-0.88	0.87	0.77-0.93	0.79	0.66-0.88	0.81	0.67-0.89	0.79	0.66-0.88
MVPA in the usual week	0.74	0.64-0.82	0.76	0.60-0.86	0.73	0.57-0.83	0.77	0.61-0.87	0.71	0.54-0.82
VPA frequency	0.68	0.55-0.77	0.73	0.56-0.85	0.61	0.41-0.76	0.72	0.54-0.84	0.62	0.42-0.76
VPA time per week	0.57	0.42-0.66	0.66	0.45-0.80	0.50	0.27-0.68	0.58	0.35-0.75	0.56	0.34-0.72
***Sedentary Behaviour***										
Watching TV on school days	0.72	0.61-0.81	0.91*	0.83-0.95	0.51	0.28-0.69	0.86^†^	0.76-0.92	0.57	0.36-0.73
Watching TV at weekends	0.74	0.63-0.83	0.77	0.62-0.87	0.70	0.53-0.82	0.80	0.66-0.89	0.68	0.49-0.80
Playing PC or console games on school days	0.54	0.38-0.67	0.56	0.32-0.73	0.53	0.30-0.70	0.56	0.32-0.73	0.52	0.29-0.70
Playing PC or console games at weekends	0.69	0.57-0.78	0.47*	0.20-0.67	0.83	0.72-0.90	0.79	0.64-0.88	0.57	0.35-0.73
Using PC on school days	0.33	0.14-0.50	0.19	-0.11-0.46	0.45	0.20-0.64	0.38	0.10-0.60	0.28	0.01-0.51
Using PC at weekends	0.50	0.33-0.64	0.37	0.09-0.60	0.58	0.36-0.73	0.83^†^	0.71-0.90	0.16	-0.12-0.41
Doing homework on school days	0.78	0.68-0.85	0.81	0.68-0.89	0.74	0.59-0.85	0.75	0.59-0.86	0.73	0.57-0.84
Doing homework at weekends	0.73	0.62-0.82	0.69	0.49-0.82	0.76	0.62-0.86	0.54	0.29-0.72	0.79	0.65-0.87
***Sleep***										
When go to bed on school days	0.75	0.65-0.83	0.76	0.60-0.86	0.73	0.57-0.84	0.68	0.48-0.81	0.60	0.39-0.75
When go to bed at weekends	0.64	0.50-0.67	0.64	0.43-0.79	0.62	0.41-0.76	0.51	0.26-0.70	0.58	0.36-0.74
When wake up on school mornings	0.77	0.68-0.84	0.79	0.65-0.88	0.76	0.62-0.86	0.81	0.68-0.89	0.73	0.57-0.84
When wake up at weekends	0.83	0.75-0.88	0.82	0.69-0.90	0.84	0.73-0.90	0.85	0.74-0.92	0.78	0.65-0.87

ICC = Single measure intraclass correlation coefficient

HBSC = Health Behaviour in School-aged Children

* Significant difference between genders (p < 0.05)

^† ^Significant difference between age groups (p < 0.05)

**Table 5:** ICC for HBSC survey questionnaire items about substance use of 15-year-old children by gender (n = 51)

	All (n = 51)		Girls (n = 20)		Boys (n = 31)	
			
	ICC	95% CI	ICC	95% CI	ICC	95% CI
Have ever smoked	0.75	0.60-0.85	NA	/	0.63	0.36-0.80
How often smoke at present	0.50	0.27-0.68	NA	/	0.48	0.15-0.71
How many cigarettes ever smoked	0.85	0.75-0.91	NA	/	0.81	0.64-0.90
How often drink beer	0.80	0.67-0.88	0.96	0.90-0.98	0.82	0.66-0.91
How often drink wine	0.53	0.30-0.70	0.70	0.39-0.87	0.42	0.09-0.67
How often drink strong liquors	0.44	0.19-0.64	0.76	0.49-0.90	0.64	0.37-0.81
Have ever been drunk	0.85	0.76-0.91	0.83	0.61-0.93	0.85	0.72-0.93

ICC = Single measure intraclass correlation coefficient

HBSC = Health Behaviour in School-aged Children

NA: Not applicable due to lack of variance

### Physical activity

The reliability of the four items assessing Moderate to Vigorous Physical Activity (MPVA) and Vigorous Physical Activity (VPA) ranged from moderate (ICC = 0.57) to almost perfect agreement (ICC = 0.82) in general. The lowest reliability was found in the item measuring VPA time per week and the highest reliability in the item relating to MVPA in the last 7 days. No statistically significant differences were found either by gender or by age group, though the ICC value may differ.

### Sedentary behaviour

Of the eight items examining the sedentary behaviours, seven of them showed a moderate to a substantial agreement. The question inquiring about using a computer on school days was the only item which indicated a fair agreement, and expressed the lowest value of ICC (0.33) for all respondents among all the selected items in this study. Significant gender differences were found in items on watching TV on school days and playing computer or console games at weekends (p < 0.05). Meanwhile, significant age differences were found in items on watching TV on school days and using a computer at weekends (p < 0.05).

### Sleep

All items on sleep patterns demonstrated at least substantial reliability, especially for the item on when children wake up at weekends, for which the reliability is almost perfect (ICC = 0.83). On the contrary, the lowest value of ICC was found for the item on when children go to bed at weekends (ICC = 0.64). There were no gender and age differences in these items.

### Substance use

The items on substance use were evaluated only for students aged 15 years old. Four items indicated a substantial to almost perfect reliability and the values of ICC varied from 0.75 to 0.85. The other three items showed at least moderate reliability and the lowest reliability was exhibited by the question of how often do you drink strong liquors (ICC = 0.44). None of the girls in this study reported they have ever smoked, so this constant result lead to the value of ICC for three items on smoking not applicable due to lack of variance.

## Discussion

Overall, the test-retest reliability results showed moderate to almost perfect agreement for most of the items, except for one item about sedentary behaviour. Findings in our study suggest that these indicators are reliable to measure health behaviour of school-aged children in Beijing. A few gender and age group differences were observed in the reliability of some indicators measuring sedentary behaviour among respondents.

The reliability of items measuring physical activity in this study indicated that both MVPA and VPA items are reliable measures of physical activity, which is a similar finding compared to previous studies [23,27,29,36,37]. One interesting finding from our study was that the lowest reliability was found for the item measuring VPA time per week (ICC = 0.57), whereas usually VPA is more easily recalled than MVPA in adults. One possible reason for this might be that young people are in a period of trying different new sports and exercise. Therefore, compared to VPA, MVPA on a daily basis is more stable, although it is more difficult to recall. Vuori and his colleagues also reported similar results concerning the test-retest reliability of HBSC survey items measuring MVPA and VPA [29]. When considering items measuring physical activity, another interesting observation was that no age group differences were found in our study whereas some earlier studies have reported that the reliability of self-reported physical activity indicators generally improve with age [27,28,37]. However, it should be noted that the lack of age effects could partly reflect low statistical power to detect differences in coefficients. In addition, gender differences were not found in this study, unlike the findings of Rangul and his colleagues in their study [28], which showed items about physical activity in the HBSC questionnaire were more reliable for girls. A possible explanation for the non-existent difference within gender and age groups may be the fact that since 2007 the 'Sunshine Project' was carried out in all primary schools and high schools in China to ensure each student participates in physical activity at least one hour per day. This results in the students having a clear consciousness concerning physical activity participation so that the behaviour can be reported accurately no matter the age and gender. However this conclusion should be viewed with caution since the sample size of this study is rather small.

Similarly to the earlier study of Hardy and his colleagues [38], the items about sedentary behaviour in this study showed acceptable reliability. However, the reliability of items related to sedentary behaviour is lower than other behaviours. A striking result is that the item on "using a computer on school days" showed the lowest value of ICC (0.33) in all selected questions. One possible reason for this finding is that students probably do not have the same possibility to access the computer at school on school days because of the different school curriculum and content of study in different school weeks. In general, the reasons for the low value of ICC are mainly due to poor reliability of answering the item or the behaviour which the item measured is not very stable between the test and retest. For this item, the poor agreement was most likely due to the rather unstable behaviour caused by the school schedule which influenced the students' use of the computer on school days. The results also revealed a difference between age and gender groups, younger students and girls tended to be more reliable than older students and boys for several items on sedentary behaviour. One exception that should be pointed out is for the item inquiring about "playing computer or console games at weekends", boys are more reliable than girls probably because playing computer or console games is predominately a boys' activity, and girls' value is different, so that they might report inaccurately.

Normally, for the self-report measures, the more response alternatives used, the more reliability is found. It is not surprising that at least substantial reliability was revealed in questions asking about sleeping habits since at least seven to fifteen response alternatives were recruited for them. Added to that, since sleep is a regular daily activity, knowledge and salience of sleep would be high. These results were very similar to the findings of Tynjälä's study [32]. It is evident for students that they have to wake up at a certain time in order to attend school on school days. Consequently, the items measuring sleeping behaviour are stable to some extent.

The study showed that items relating to smoking and alcohol use for 15 year-old students have a good reliability which is not surprising, as the finding is similar to previous studies [39,40]. An explanation for this is the fact that substance use displays a certain degree of cross-time stability, and therefore it can be recalled more reliably than other health behaviours [41]. In addition, the salience of smoking and alcohol use might be higher compared to other health behaviours, since most students need to an attitude towards such behaviours. Normally smoking behaviour would not change in the short term, but considering the students smoking is absolutely prohibited in Chinese schools and by most of their parents, it is understandable that the present smoking frequency of students who smoked may differ in terms of the different possibility to access cigarettes and smoke them. Another notable finding is that when students were asked about how often they drink beer, wine and strong liquors, the answers for wine and strong liquors are not as stable as for beer. The underlying reason for this is that many students have no clear definition of wine and strong liquors because compared to western countries, wine is rather seldom drunk for the masses in China, and the diversity of Chinese strong liquors makes students' recall consumption unreliably compared to beer. Accordingly, these two items should be considered for revision or addition of more reference explanations.

As a part of the pilot study for the Health Behaviour and Lifestyle Survey for School-aged Children in Beijing 2008, the test-retest study was conducted during the normal school class. None of the students in the sample classes refused to fill in the questionnaire and all respondents could complete the questionnaire within one school hour (45 minutes). No questions or more interpretations were asked about the items used in the questionnaire during the data collection. Those indicators measuring health behaviour in the survey questionnaire proved to be understandable and acceptable to the school-aged children in Beijing.

Although it is the first assessment of the test-retest reliability of items related to several indicators measuring health behaviour used in the HBSC survey questionnaire in a Chinese population, this study has several limitations. First, the sample size for the test-retest study is small and the two sampled schools both come from the urban area of Beijing. For a country like China, when social economic status and culture background are taken into account, it is challenging to interpret the findings without a large and diverse sample. Second, reliability is a necessary characteristic of a valid self-report measure, but it is not sufficient to ensure the validity of questions. This study, however, did not examine the validity of survey indicators. Furthermore, qualitative study on the acceptability and reproducibility of the HBSC survey questionnaire is lacking in our study. Finally, to support using the HBSC survey questionnaire in a Chinese population, and in a future possible China HBSC study, more work should be encouraged to assess both reliability and validity of the HBSC survey questions among Chinese adolescents.

## Conclusions

This study represents the first assessment of the test-retest reliability of items, concerning physical activity, sedentary behaviour, sleep and substance use, from the HBSC survey questionnaire, in a Chinese population. The overall findings of this study suggest that most selected items in the HBSC survey questionnaire have satisfactory test-retest reliability for school-aged children in Beijing urban area. Despite the limitations, this study provided valuable information on feasibility and reliability of the HBSC survey questionnaire for the school-aged children in Beijing urban area. Further studies in larger and more diverse samples, as well as validity studies should be considered in both urban and rural areas for the future Chinese HBSC study.

## Competing interests

The authors declare that they have no competing interests.

## Authors' contributions

All authors have read and approved the manuscript. YL, first author, made a substantial contribution to analyze data and write the original manuscript. MW contributed leading and designing the data collection, discussions and comments on the draft. JT and LK commented and revised the draft throughout the whole writing process. YL and ZZ participated in collecting data and commented on the draft. JV contributed by giving statistical support and commenting on the draft.

## Pre-publication history

The pre-publication history for this paper can be accessed here:

http://www.biomedcentral.com/1471-2288/10/73/prepub
